# Cytokinesis‐Defective 1 (CYT1) Positively Regulates Plant Antiviral Immunity by Promoting Callose Deposition and Ascorbic Acid Biosynthesis

**DOI:** 10.1111/mpp.70126

**Published:** 2025-07-09

**Authors:** Xue Jiang, Yuting Wang, Yu Zhao, Xayvangye Korxeelor, Wenqian Fan, Xinyue Fan, Yong Li, Xiaoxia Wu, Xueping Zhou, Fangfang Li, Xiaoyun Wu, Weiqin Ji, Xiaofei Cheng

**Affiliations:** ^1^ College of Plant Protection Northeast Agricultural University Harbin Heilongjiang China; ^2^ School of Life Science Northeast Agricultural University Harbin Heilongjiang China; ^3^ College of Agriculture Northeast Agricultural University Harbin Heilongjiang China; ^4^ State Key Laboratory for Biology of Plant Diseases and Insect Pests, Institute of Plant Protection Chinese Academy of Agricultural Sciences Beijing China

**Keywords:** ascorbic acid, callose, CYT1, NIb, TuMV

## Abstract

NUCLEAR INCLUSION B (NIb), the RNA‐dependent RNA polymerase (RdRp) of potyviruses, plays a critical role in both viral replication and suppression of host antiviral immunity. However, the mechanisms by which NIb suppresses host immunity remain poorly understood. In this study, we used affinity purification‐mass spectrometry to identify host factors interacting with NIb encoded by turnip mosaic virus (TuMV). We identified 57 potential NIb‐interacting host factors, including mannose‐1‐phosphate guanylyltransferase CYTOKINESIS‐DEFECTIVE 1 (CYT1). Virus infectivity assays showed that TuMV infection was significantly attenuated in *Nicotiana benthamiana* leaves transiently expressing *CYT1* and in transgenic *Arabidopsis* overexpressing *CYT1*. Exogenous application of ascorbic acid (AsA) and inhibition of *N*‐linked glycosylation reduced virus infection. Furthermore, overexpression of *CYT1* induced callose deposition, and inhibition of callose synthesis enhanced virus infection. We also demonstrated that NIb interacts with the C‐terminal domain of CYT1, affects its cytosolic distribution, and inhibits AsA accumulation. These findings suggest that CYT1 positively regulates plant antiviral immunity by promoting callose deposition and ascorbic acid biosynthesis.

## Graphical abstract

Turnip mosaic virus (TuMV)‐encoded NIb protein interacts with CYT1 to reduce callose deposition and ascorbic acid biosynthesis, while promoting *N*‐linked glycosylation to enable robust infection.

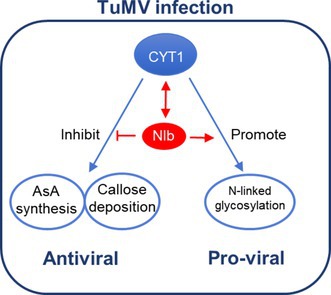

## 1 Introduction

Vitamin C (l‐ascorbic acid; AsA) is a well‐known plant metabolite that plays a crucial role in plant cell division and elongation as a cofactor for enzymes involved in biosynthesis of phytohormones such as ethylene, gibberellic acid (GA), or abscisic acid (ABA) (Gallie 2013). As an antioxidant, AsA helps plants respond to abiotic and biotic stresses by detoxifying reactive oxygen species (ROS), including singlet oxygen (^1^O_2_), superoxide anion (O_2_˙^−^), hydroxyl radical (OH˙), and hydrogen peroxide (H_2_O_2_). For example, high‐dose AsA treatment has been shown to alleviate symptoms caused by cucumber mosaic virus (CMV), turnip crinkle virus (TCV), and tobacco necrosis virus (TNV), and inhibits virus accumulation at later infection stages (Wang et al. 2011). In plants, AsA is primarily biosynthesised via the Smirnoff–Wheeler pathway, although three additional minor pathways also contribute (Gallie 2013). *CYTOKINESIS‐DEFECTIVE 1* (*CYT1*), also known as *VITAMIN C‐DEFECTIVE 1* (*VTC1*), encodes mannose‐1‐phosphate guanylyl transferase, which catalyses the conversion of α‐d‐mannose 1‐phosphate to GDP‐α‐d‐mannose in the Smirnoff–Wheeler pathway (Conklin et al. 1999). Interestingly, GDP‐α‐d‐mannose is also required for *N*‐linked glycosylation of cellulose and proteins (Nickle and Meinke 1998). Consequently, a null mutant of *CYT1* is embryonically lethal and early embryos exhibit incomplete cell walls and excessive callose deposition (Nickle and Meinke 1998). Similarly, the *vtc1* mutant, which has a cytosine‐to‐thymine substitution resulting in a proline‐to‐serine change at amino acid position 22 of CYT1, accumulates only about 25% of AsA compared to wild‐type plants and shows increased resistance to the bacterial pathogen *Pseudomonas syringae* (Fujiwara et al. 2016; Mukherjee et al. 2010; Barth et al. 2004). These findings suggest that reduced AsA levels may lead to higher ROS accumulation, thereby increasing resistance. However, the precise role of AsA and CYT1 in plant immunity remains unclear.

Turnip mosaic virus (TuMV) is a typical member of the genus *Potyvirus* in the family *Potyviridae* (Walsh and Jenner 2002). The TuMV genome consists of a single‐stranded, positive‐sense RNA (+ssRNA) molecule encapsulated in non‐enveloped, flexuous, filamentous viral particles of approximately 720 nm length and 13–15 nm in diameter. The +ssRNA genome encodes a single large open reading frame (ORF) that is translated into a large polyprotein, which is subsequently cleaved by three viral proteases into 10 mature proteins, FIRST PROTEIN (P1), HELPER‐COMPONENT PROTEASE (HcPro), THIRD PROTEIN (P3), 6‐KILODALTON PEPTIDE 1 (6K1), CYLINDRICAL INCLUSION (CI), 6‐KILODALTON PEPTIDE 2 (6K2), VIRAL PROTEIN GENOME‐LINKED (VPg), NUCLEAR INCLUSION PROTEIN A PROTEASE (NIa‐Pro), NUCLEAR INCLUSION PROTEIN B (NIb), and COAT PROTEIN (CP) (Walsh and Jenner 2002). Additionally, a polymerase slippage motif in the P3 cistron allows the expression of a short polyprotein that is cleaved into P1, HcPro, and AMINO‐TERMINAL HALF OF P3 FUSED TO PIPO (P3N‐PIPO) (Chung et al. 2008). A recent study has identified novel ORFs in the minus strand of some RNA viruses, including TuMV (Gong et al. 2023). Interestingly, TuMV infection induces AsA accumulation in *Brassica rapa* , but the resistances of *B. rapa* cultivars to TuMV is negatively correlated with AsA and its dehydrogenated product, dehydroascorbic acid (DHA) levels (Fujiwara et al. 2016). Furthermore, *vtc1* plants were more susceptible to TuMV than wild‐type plants (Fujiwara et al. 2016), suggesting a potential antiviral role for CYT1. However, the exact role of the AsA pathway in limiting TuMV infection and how the virus counteracts this resistance remain unclear. In this study, we report the interaction between TuMV‐encoded NIb and CYT1 and explore the antiviral function of CYT1.

## 2 Results

### 2.1 TuMV NIb Interacts With CYT1

**FIGURE 1:**
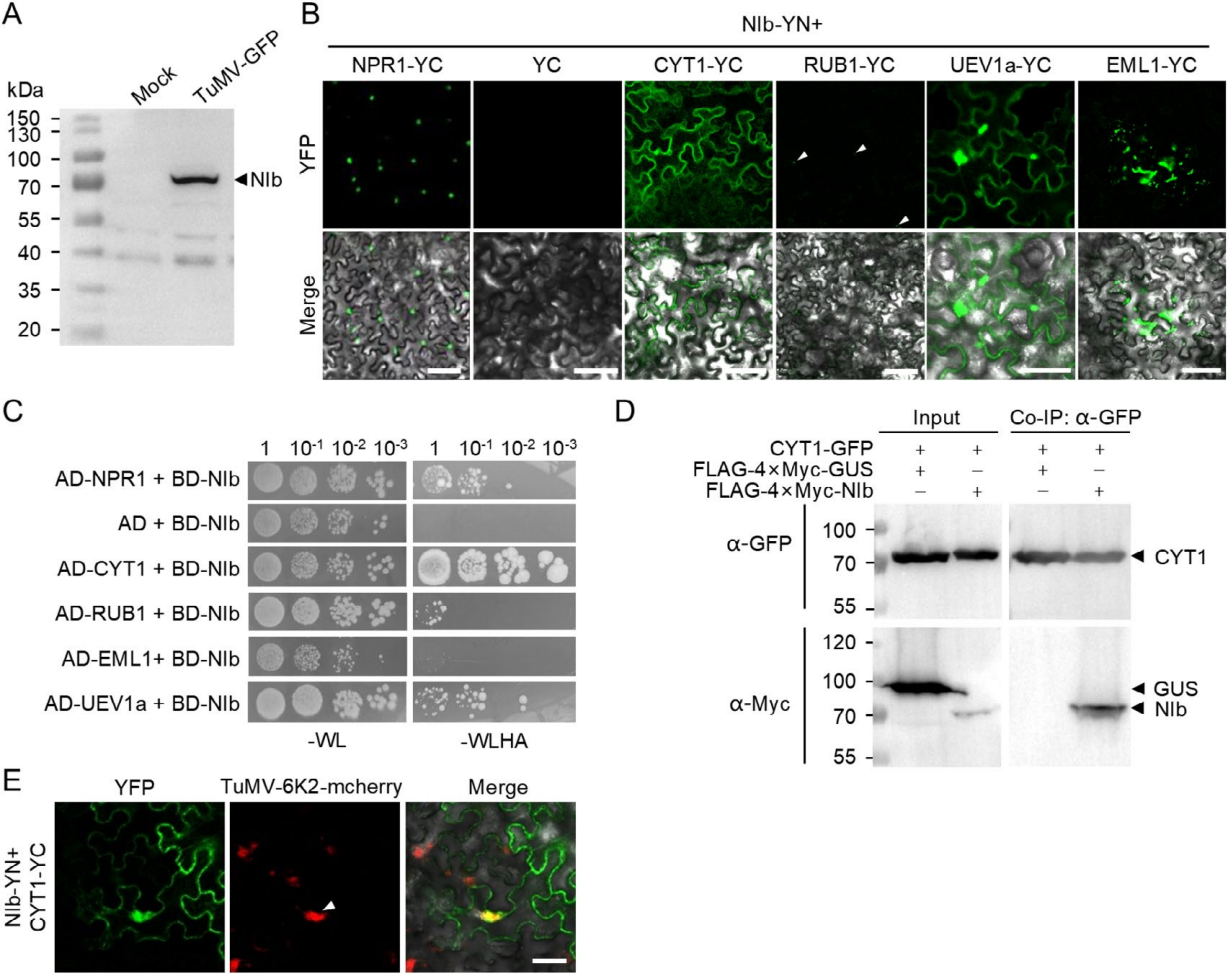
TuMV NIb interacts with CYT1. (A) Western blot analysis of affinity‐purified NIb protein from TuMV‐GFP‐infected *Arabidopsis thaliana* seedlings using anti‐NIb antibodies. The position of NIb is indicated. (B) Confocal microscopy images of *Nicotiana benthamiana* epidermal cells expressing the indicated plasmids at 2 days post‐infiltration (dpi). Scale bar: 50 μm. White arrowheads indicate small fluorescent foci. All images were acquired under identical settings. (C) Growth of serially diluted yeast cells transformed with BD‐NIb and AD‐tagged proteins (NPR1, CYT1, RUB1, EML1, and UEV1a) on synthetic defined (SD) medium lacking tryptophan and leucine (–WL) or lacking tryptophan, leucine, histidine, and adenine (–WLHA). (D) Co‐immunoprecipitation (Co‐IP) assay validating the interaction between NIb and CYT1. CYT1‐GFP was coexpressed with FLAG‐4 × Myc‐NIb or FLAG‐4 × Myc‐GUS (control) in *N. benthamiana* leaves via agroinfiltration. Proteins were immunoprecipitated with GFP‐trap agarose at 2 dpi and detected using anti‐GFP or anti‐Myc antibodies. (E) Confocal microscopy images of TuMV‐6K2mCherry‐infected *N. benthamiana* epidermal cells infiltrated with agrobacteria harbouring NIb‐YN and CYT1‐YC at 2 dpi. Scale bar: 50 μm. White arrowhead indicates 6K2‐mCherry‐labelled viral replication complex.

To identify host factors interacting with NIb during TuMV infection, we inoculated 3‐week‐old wild‐type *Arabidopsis thaliana* ecotype Coloumbia‐0 (Col‐0) seedlings by agroinfiltration with TuMV‐GFP, a TuMV infectious clone expressing GFP between the P1 and HcPro cistrons (Lellis et al. 2002). Two weeks post‐inoculation, virus‐infected leaf tissue was harvested for NIb purification using agarose covalently linked to polyclonal antibodies against NIb. A parallel affinity purification was performed using mock‐treated *A. thaliana* plants of the same age as a control. Western blotting confirmed NIb purification (Figure 1A). The purified proteins were analysed by tandem mass spectrometry (MS/MS) in duplicate, and the resulting peptides were searched against the TuMV‐GFP‐encoded peptides. Peptides from the two TuMV‐infected plant samples covered 46% and 26% of NIb, respectively, while no peptide in the two mock samples matched NIb (Table S1). Several viral proteins, including CI, P3, HcPro, NIa‐Pro and CP, were also identified in both TuMV‐infected plant samples. The peptides were further searched against the *A. thaliana* UniProt database, and proteins present only in the TuMV‐infected plant were selected as NIb‐interacting candidates. A total of 57 NIb‐interacting host candidates were identified (Table S2). Among these, five candidates, namely, CYT1, NEXT TO BRCA1 GENE 1 (NBR1), PROTEOLYSIS 6 (PRT6), LIGHT‐HARVESTING CHLOROPHYLL B‐BINDING 2.2 (LHCB2.2), and 75 KDA TRANSLOCON AT THE OUTER‐ENVELOPE‐MEMBRANE OF CHLOROPLASTS 3 (TOC75‐3), were found in both replicates. Notably, the interaction between NBR1 and NIb has been previously confirmed (Li et al. 2020), validating the specificity of our MS/MS assay.

Four candidates, namely CYT1, EMSY‐LIKE 1 (EML1), UBIQUITIN CONJUGATING ENZYME E2 VARIANT 1A (UEV1a), and UBIQUITIN‐RELATED PROTEIN 1 (RUB1), were selected for further confirmation based on their coverage, biological functions, and subcellular localisation (excluding chloroplast, mitochondrial, and vacuolar factors) (Table S2). The full‐length coding regions of these genes were amplified from wild‐type *A. thaliana* Col‐0 and coexpressed with N‐terminal domain of yellow fluorescent protein (YN)‐tagged NIb (NIb‐YN) as the C‐terminal domain of yellow fluorescent protein (YC)‐tagged recombinant proteins under the cauliflower mosaic virus (CaMV) 35S promoter in *Nicotiana benthamiana* epidermal cells. Empty YC was used as a negative control, while NONEXPRESSOR OF PATHOGENESIS‐RELATED GENES 1 (NPR1), a known NIb‐interacting protein (Liu et al. 2023), served as a positive control. At 2 days post‐infiltration (dpi), bright yellow fluorescence was observed in the cells coexpressing NIb‐YN with NPR1‐YC, CYT1‐YC, UEV1a‐YC, and EML1‐YC, while small fluorescent foci were observed in the cells coexpressing NIb‐YN and RUB1‐YC (Figure 1B). Yeast two‐hybrid (Y2H) assays further confirmed these interactions, with yeast cells transformed with BD‐NIb plus AD‐NPR1, AD‐CYT1, and UEV1a growing well on selective medium, while those transformed with BD‐NIb plus AD‐RUB1 showed slight growth, BD‐NIb plus AD‐EML1 failed to grow (Figure 1C). These results suggest that NIb strongly interacts with CYT1 and UEV1a, and weakly with RUB1. Given CYT1's role in AsA synthesis and *N*‐linked glycosylation, we focused on CYT1 for further study. Coimmunoprecipitation (CoIP) confirmed the interaction between NIb and CYT1, with CYT1‐GFP coprecipitating with FLAG‐4 × Myc‐NIb but not with FLAG‐4 × Myc‐GUS (Figure 1D). To further confirm the interaction during virus infection, we performed a bimolecular fluorescence complementation (BiFC) assay in the epidermal cells infected by TuMV‐6K2mCherry. Bright YFP signal from the interaction between NIb and CYT1 was recorded in the cytoplasm. Interestingly, strong YFP signal was found in the viral replication complexes (VRCs) that were labelled by 6K2‐mCherry (Figure 1E). These findings demonstrate that CYT1 is a TuMV NIb‐interacting host factor.

### 2.2 CYT1 Plays an Antiviral Role

**FIGURE 2:**
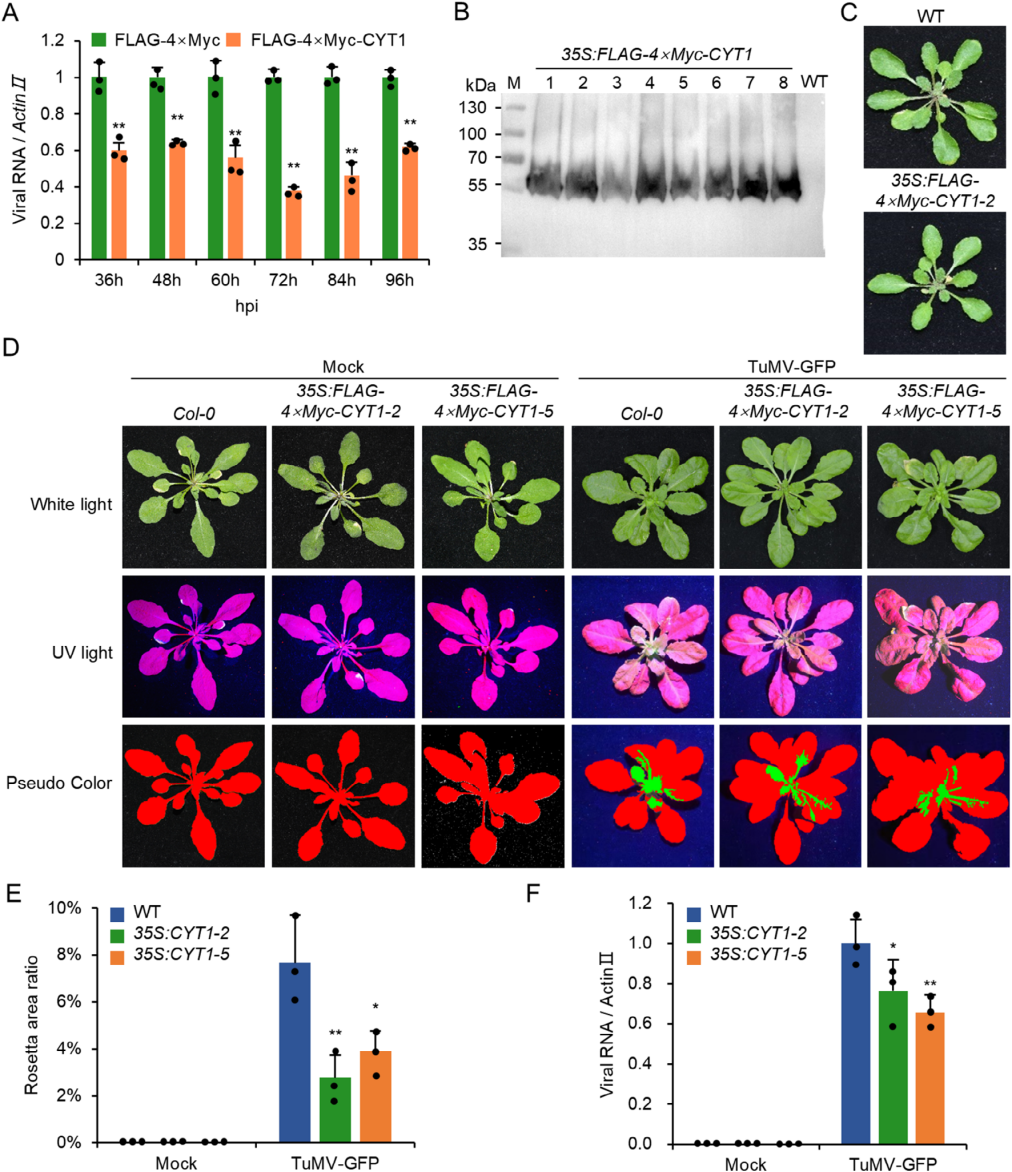
Antiviral activity of CYT1. (A) Bar graph showing the accumulation of TuMV‐GFP genomic RNA in *Nicotiana benthamiana* leaves expressing FLAG‐4 × Myc or FLAG‐4 × Myc‐CYT1 at indicated time points (*n* = 3). Reverse transcription‐quantitative PCR (RT‐qPCR) was performed using *Actin II* as an internal control, with viral genome levels in FLAG‐4 × Myc‐expressing leaves normalised to 1. (B) Immunoblot analysis of FLAG‐4 × Myc‐CYT1 expression in transgenic *Arabidopsis thaliana* . (C) Phenotypes of 4‐week‐old wild‐type (WT) and *35S:FLAG‐4 × Myc‐CYT1‐2* plants under steady‐state conditions. (D) Phenotypes of WT and transgenic *A. thaliana* plants infiltrated with empty agrobacteria (mock) or *Agrobacterium* harbouring TuMV‐GFP under white light and ultraviolet (UV) illumination at 14 dpi. Pseudocolor panels indicate virus‐free (red) and virus‐infected (green) areas. (E) Bar chart showing the ratio of TuMV‐infected to the total leaf area in WT and transgenic plants at 14 days post‐infiltration (dpi) (*n* = 3). (F) Bar graph of relative viral genome levels in WT and transgenic plants at 14 dpi (*n* = 3). RT‐qPCR data were normalised to *Actin II*, with viral genome levels in WT set to 1. In all panels, * and ** indicate *p* ≤ 0.05 and 0.01, respectively (Student's *t* test).

To analyse CYT1's role in TuMV replication, we transiently expressed FLAG‐4 × Myc‐tagged CYT1 (FLAG‐4 × Myc‐CYT1) or the FLAG‐4 × Myc tag alone with TuMV‐GFP in *N. benthamiana* leaves under the CaMV 35S promoter. Reverse transcription‐quantitative PCR (RT‐qPCR) analysis from 36 to 96 h post‐inoculation (hpi) at 12 h intervals showed that TuMV genome accumulation was significantly reduced in cells co‐expressing FLAG‐4 × Myc‐CYT1 compared to cells expressing the FLAG‐4 × Myc tag alone (Figure 2A), suggesting an antiviral function for CYT1. To confirm this, we generated transgenic *Arabidopsis* overexpressing FLAG‐4 × Myc‐CYT1 under the CaMV 35S promoter (*35S:FLAG‐4 × Myc‐CYT1*). Eight independent transgenic lines were obtained, with western blotting confirming FLAG‐4 × Myc‐CYT1 expression in all lines (Figure 2B). Transgenic plants exhibited normal phenotypes under steady‐state conditions (Figure 2C). Three‐week‐old seedlings of wild‐type and two transgenic lines (*35S:FLAG‐4 × Myc‐CYT1‐2* and *35S:FLAG‐4 × Myc‐CYT1‐5*) were mechanically inoculated with TuMV‐GFP. At 14 dpi, the virus‐infected leaf area in transgenic plants was significantly reduced compared to wild‐type plants (Figure 2D,E). RT‐qPCR confirmed lower TuMV genome accumulation in transgenic plants (Figure 2F). To further confirm the antiviral function of CYT1, we infiltrated *N. benthamiana* with TuMV‐GFP/ΔCP, a movement‐defective TuMV mutant containing a stop codon in the CP cistron (Dai et al. 2020), and then treated by 10 or 50 mM AsA. At 5 dpi, the accumulation of viral RNA was analysed by RT‐qPCR. The results showed that AsA treatment significantly inhibited the accumulation of viral RNA (Figure S1). Taken together, these results indicate that CYT1 has an antiviral function.

### 2.3 NIb Interacts With the C‐Terminal Domain of CYT1 and Reduces AsA Accumulation

**FIGURE 3:**
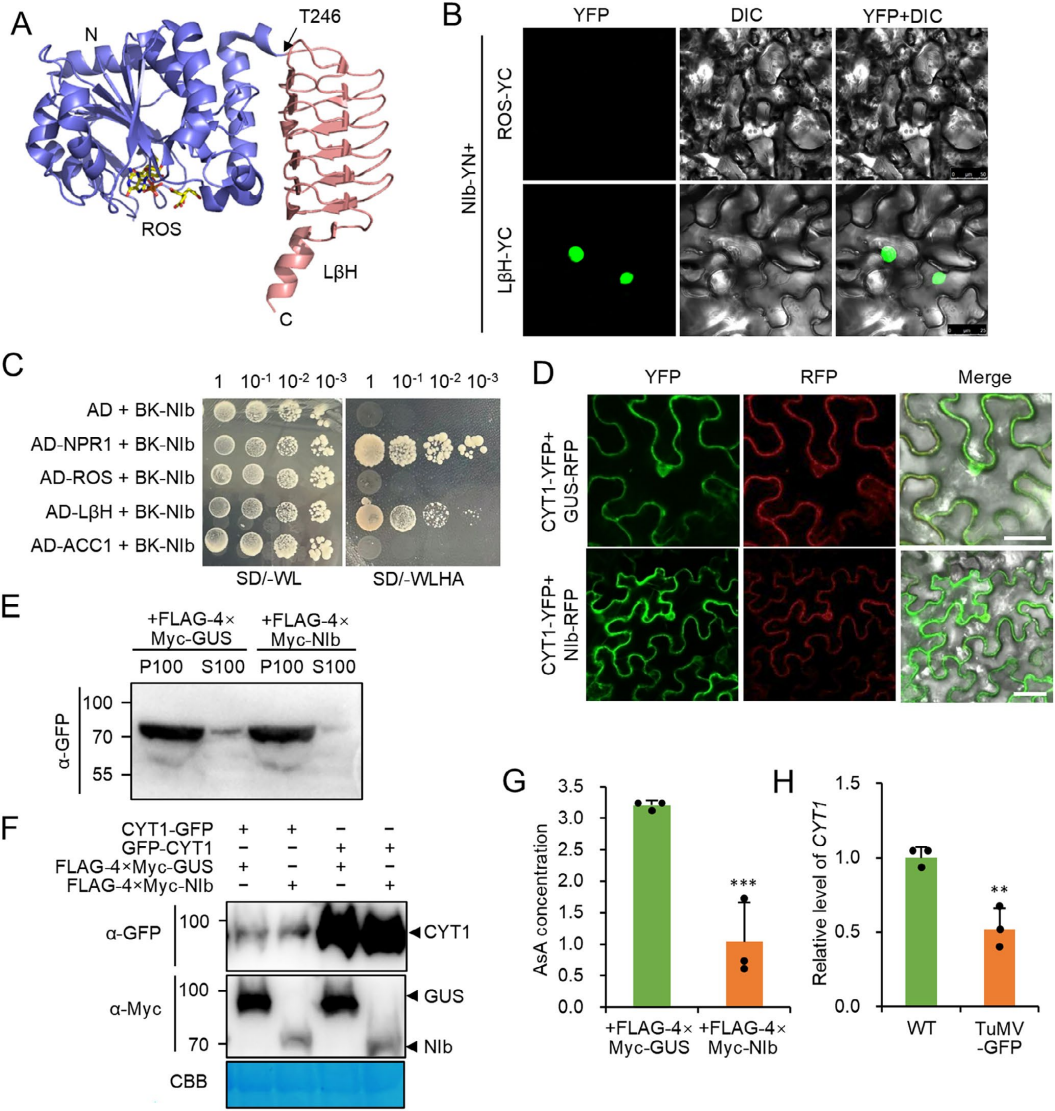
TuMV NIb interferes with the CYT1‐mediated ascorbic acid (AsA) metabolic pathway. (A) Cartoon representation of CYT1. The N‐terminal Rossmann fold‐like catalytic (ROS) domain (light blue) and C‐terminal left‐handed parallel β‐helix (LβH) domain (light pink) are shown. ROS‐bound adenosine triphosphate (ATP) is shown as stick model (yellow). Threonine 246 (T246) is marked with an arrow. N‐ and C‐terminal ends are labelled. (B) Confocal microscopy images of *Nicotiana benthamiana* epidermal cells expressing NIb‐YN with YC‐tagged ROS domain (ROS‐YC) or LβH domain (LβH‐YC) at 2 days post‐infiltration (dpi). Scale bar: 50 μm. Images were acquired under identical settings. (C) Growth of serially diluted yeast cells transformed with indicated plasmids on selective medium. (D) Confocal microscopy images of *N. benthamiana* epidermal cells coexpressing CYT1‐YFP and NIb‐RFP or GUS‐RFP (control) at 2 dpi. Scale bar: 50 μm. (E) Western blot analysis of pellets (P) and supernatants (S) after 100,000 *g* centrifugation, probed with anti‐GFP and anti‐Myc antibodies. (F) Immunoblot of CYT1‐YFP and YFP‐CYT1 accumulation in the presence of FLAG‐4 × Myc‐NIb or FLAG‐4 × Myc‐GUS (control). CBB, Coomassie Briliant Blue staining of loading control. (G) Bar graph showing AsA concentrations in *N. benthamiana* leaves expressing FLAG‐4 × Myc‐NIb or FLAG‐4 × Myc‐GUS after 12 h flg22 treatment. (H) Reverse transcription‐quantitative PCR analysis of *CYT1* expression. *CYT1* expression normalised to *AtEF1α* in wild‐type (WT) plants (*n* = 3). In all panels, ** and *** indicate *p* ≤ 0.01 and 0.001, respectively (Student's *t* test).

Based on the recently published structure of CYT1 (Zhang et al. 2022), we divided CYT1 into two domains, namely the N‐terminal Rossmann fold‐like (ROS) domain and the C‐terminal left‐handed parallel β‐helix (LβH) domain (Figure 3A). The ROS domain contains the catalytic activity, while the LβH domain may be involved in dimerisation and protein interactions (Zhang et al. 2022). BiFC assays showed bright YFP fluorescence in the nuclei of *N. benthamiana* epidermal cells expressing NIb‐YN and LβH‐YC, but no YFP fluorescence in cells expressing NIb‐YN and ROS‐YC (Figure 3B), indicating that NIb interacts with the LβH domain. Y2H assays confirmed this interaction, with yeast cells harbouring AD‐LβH and BD‐NIb growing on selective medium, while those transformed with BD‐NIb plus AD, AD‐tagged ACETYL‐COA CARBOXYLASE 1 (ACC1), which was included as a negative control, failed to grow (Figure 3C). Taken together, these results suggest that NIb interacts with the LβH domain of CYT1.

To further investigate the biological function of the NIb–CYT1 interaction, we analysed the subcellular localisation of CYT1‐YFP in *N. benthamiana* epidermal cells coexpressing NIb‐RFP or GUS‐RFP. CYT1‐YFP was mainly localised in the cytosol in both conditions (Figure 3D), suggesting that NIb may not affect CYT1's subcellular localisation. Microsomal fractionation revealed that most CYT1 was associated with the endomembrane, such as endoplasmic reticulum (ER) and plasma membrane (Figure 3E; Figure S2). Interestingly, coexpression of FLAG‐4 × Myc‐NIb reduced CYT1 levels in the cytosol (Figure 3E; Figure S2), suggesting that NIb may affect CYT1's cytosolic distribution. Western blotting showed similar levels of CYT1‐YFP or YFP‐CYT1 in cells coexpressing FLAG‐4 × Myc‐GUS or FLAG‐4 × Myc‐NIb (Figure 3F), indicating that NIb does not affect CYT1 stability as well. We also analysed the influence of CYT1 to the subcellular localisation and stability of NIb. Results showed that CYT1 had no effect on the subcellular localisation and stability of NIb as well (Figure S3). Finally, we measured AsA concentration in *N. benthamiana* leaves expressing CYT1‐YFP plus FLAG‐4 × Myc‐GUS or FLAG‐4 × Myc‐NIb. AsA levels were significantly lower in cells coexpressing FLAG‐4 × Myc‐NIb (Figure 3G), indicating that NIb inhibits AsA biosynthesis. Additionally, *CYT1* expression was significantly reduced in TuMV‐infected leaf tissue compared to mock‐treated plants (Figure 3H). Taken together, these findings suggest that NIb reduces cytosolic CYT1 levels and inhibits AsA synthesis.

### 2.4 AsA Inhibits TuMV Infection

**FIGURE 4:**
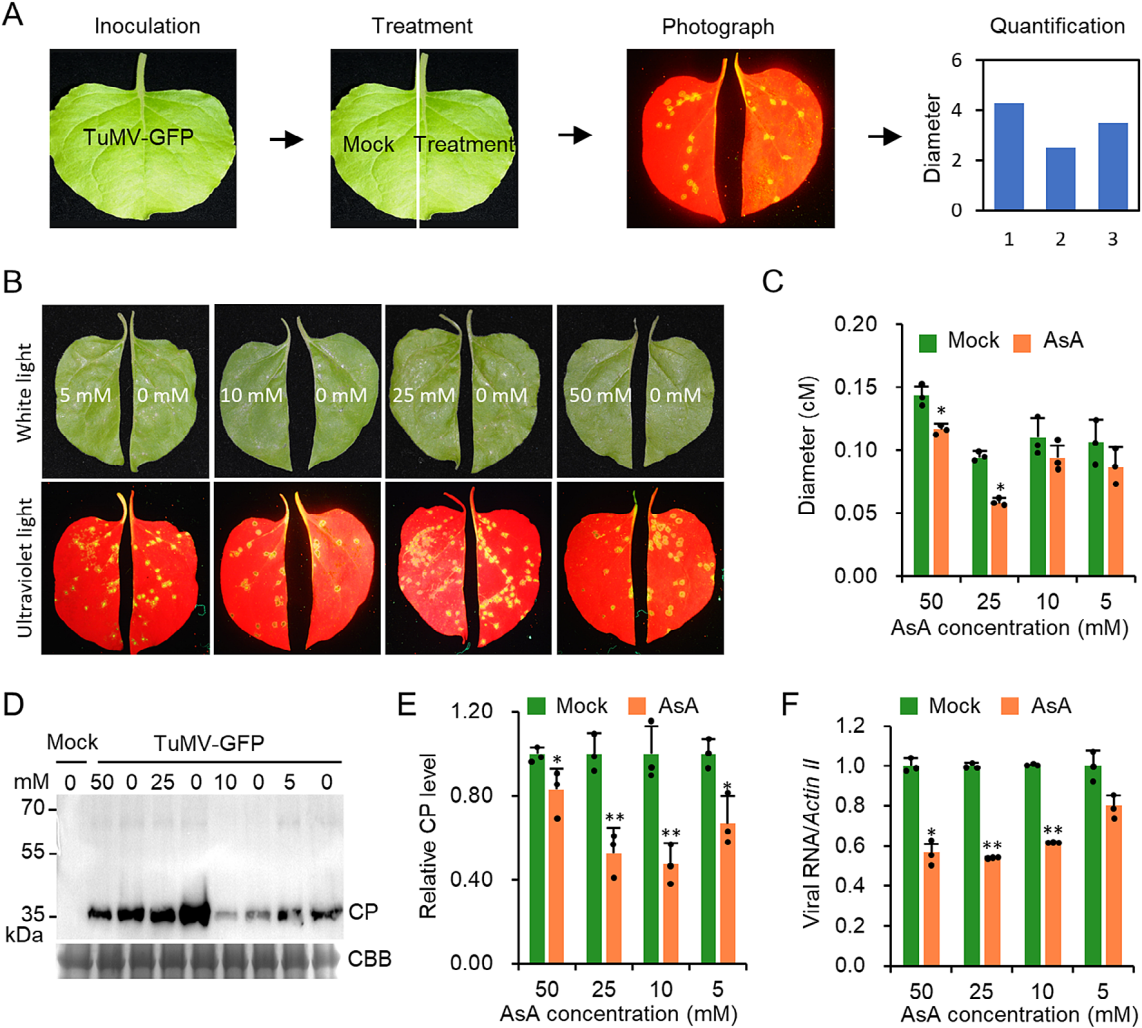
Ascorbic acid (AsA) exhibits direct antiviral activity. (A) Schematic of the in vitro virus replication inhibition assay. (B) Phenotypes of *Nicotiana benthamiana* leaves treated with indicated AsA concentrations for 5 days post‐TuMV‐GFP inoculation (dpi) under white and ultraviolet (UV) light. (C) Bar graph showing average TuMV lesions in AsA‐treated *N. benthamiana* leaves at 5 dpi (*n* = 3 half‐leaves). (D) Immunoblot of TuMV coat protein (CP) accumulation. Each treatment includes five lesions. Coomassie Brilliant Blue (CBB) staining served as the loading control. (E) Bar chart of relative CP levels normalised to mock treatment in panel (D). (F) Reverse transcription‐quantitative PCR (RT‐qPCR) analysis of viral genome levels in mock‐ and AsA‐treated samples (*n* = 3), normalised to *Actin II*. In all panels, * and ** indicate *p* ≤ 0.05 and 0.01, respectively (Student's *t* test).

*CYT1* encodes mannose‐1‐phosphate guanylyltransferase, which catalyses the conversion of α‐d‐mannose 1‐phosphate to GDP‐α‐d‐mannose in the Smirnoff–Wheeler pathway for AsA biosynthesis (Conklin et al. 1999). AsA has been reported to have direct antiviral activity (Wang et al. 2011; Fujiwara et al. 2016). We hypothesised that CYT1's antiviral function may be mediated by AsA accumulation. We developed an in vitro virus infection assay using detached *N. benthamiana* leaves (Figure 4A). *N. benthamiana* leaves were mechanically inoculated with TuMV‐GFP. One half of the leaf was treated with 5, 10, 25, and 50 mM AsA, while the other half was treated with buffer. At 5 dpi, quantification analyses showed that the size and brightness of TuMV infection foci were significantly reduced in leaves treated with 25 and 50 mM AsA compared to the control (Figure 4B,C). Western blotting and RT‐qPCR confirmed lower accumulation of TuMV CP and genomic RNA, respectively, in AsA‐treated leaves (Figure 4D–F). To further confirm the antiviral function of AsA, we analysed changes in AsA concentrations during TuMV infection at 0, 3, 6, and 12 dpi. The results showed that AsA increased significantly at 3 dpi, but there was no difference in AsA content at 6 and 12 dpi (Figure S4), indicating that TuMV infection stimulates AsA biosynthesis at the early stage of virus infection, while may be suppressed in the late stage of virus infection. Taken together, these results suggest that AsA has an antiviral role during TuMV infection.

### 2.5 *N*‐Linked Glycosylation Enhances TuMV Infection

**FIGURE 5:**
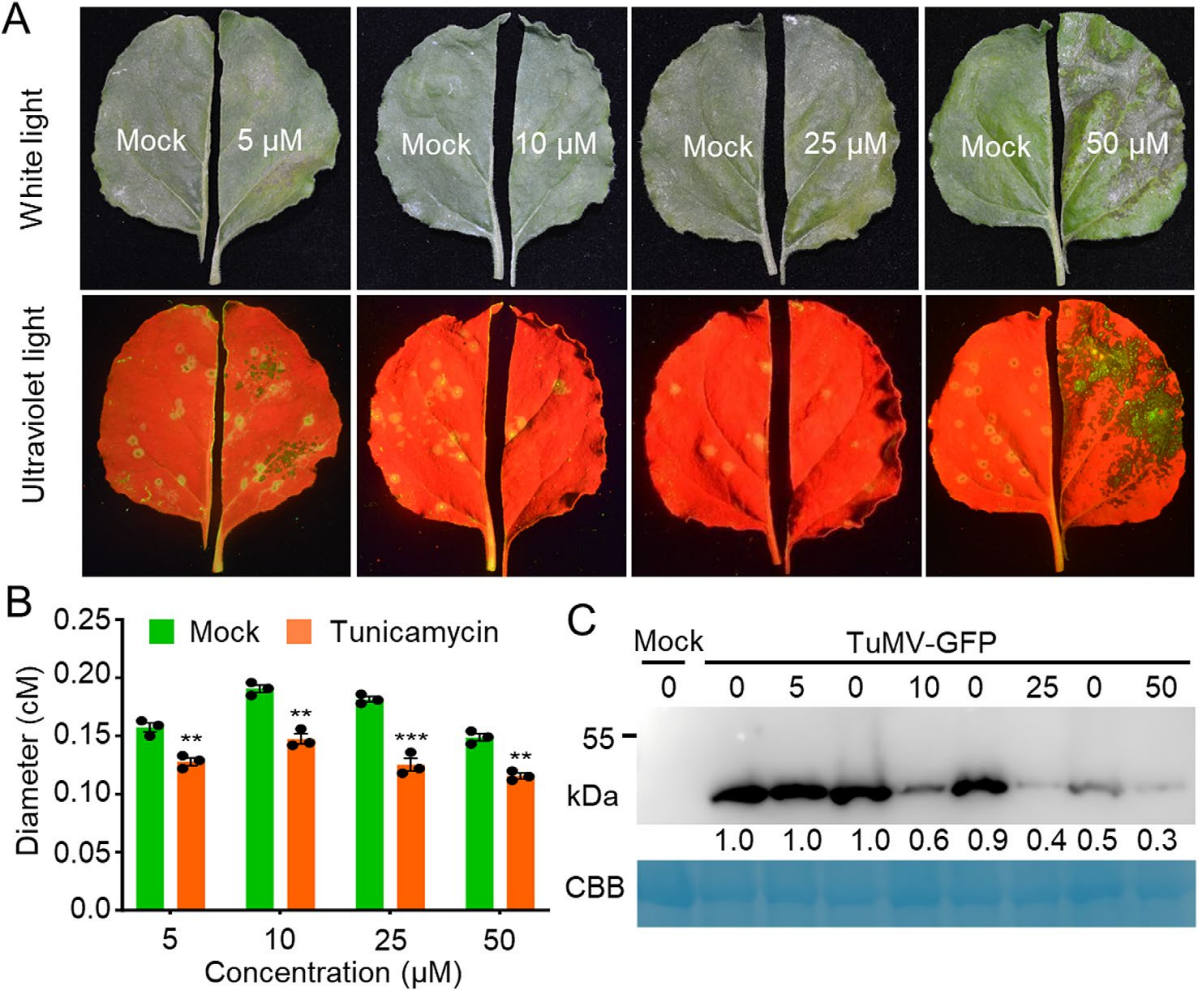
*N*‐linked glycosylation promotes turnip mosaic virus (TuMV) infection. Phenotypes of *Nicotiana benthamiana* leaves treated with indicated tunicamycin concentrations for 5 days post‐TuMV‐GFP inoculation under white and UV light. (B) Bar graph showing percentage of TuMV‐infected area (*n* = 3 half‐leaves). (C) Immunoblotting of TuMV coat protein (CP) accumulation. Each treatment includes five lesions. CBB, Coomassie Brilliant lue staining of loading control. ** and *** indicate *p* < 0.01 and *p* < 0.001, respectively (Student's *t* test).

CYT1 is also involved in *N*‐linked glycosylation of proteins and cellulose (Nickle and Meinke 1998). *N‐*linked glycosylation is essential for the infection of many animal viruses, such as dengue virus, Zika virus, influenza virus, human immunodeficiency virus, and coronaviruses (Pandey et al. 2022; Feng et al. 2022). We hypothesised that *N*‐linked glycosylation may also play a role in TuMV infection. Using the in vitro virus infection assay, we treated TuMV‐GFP‐infected *N. benthamiana* leaves with 5, 10, 25, or 50 μM tunicamycin, an inhibitor of *N*‐linked glycosylation. At 5 dpi, the virus infection foci were significantly reduced in leaves treated with 10 and 25 μM tunicamycin (Figure 5A,B). Moreover, we found that 50 μM of tunicamycin caused the necrosis of the virus‐infected area (Figure 5A). Western blotting confirmed lower TuMV CP accumulation in tunicamycin‐treated leaves (Figure 5C). These results suggest that *N*‐linked glycosylation promotes TuMV infection.

### 2.6 CYT1‐Induced Callose Deposition Inhibits TuMV Infection

**FIGURE 6:**
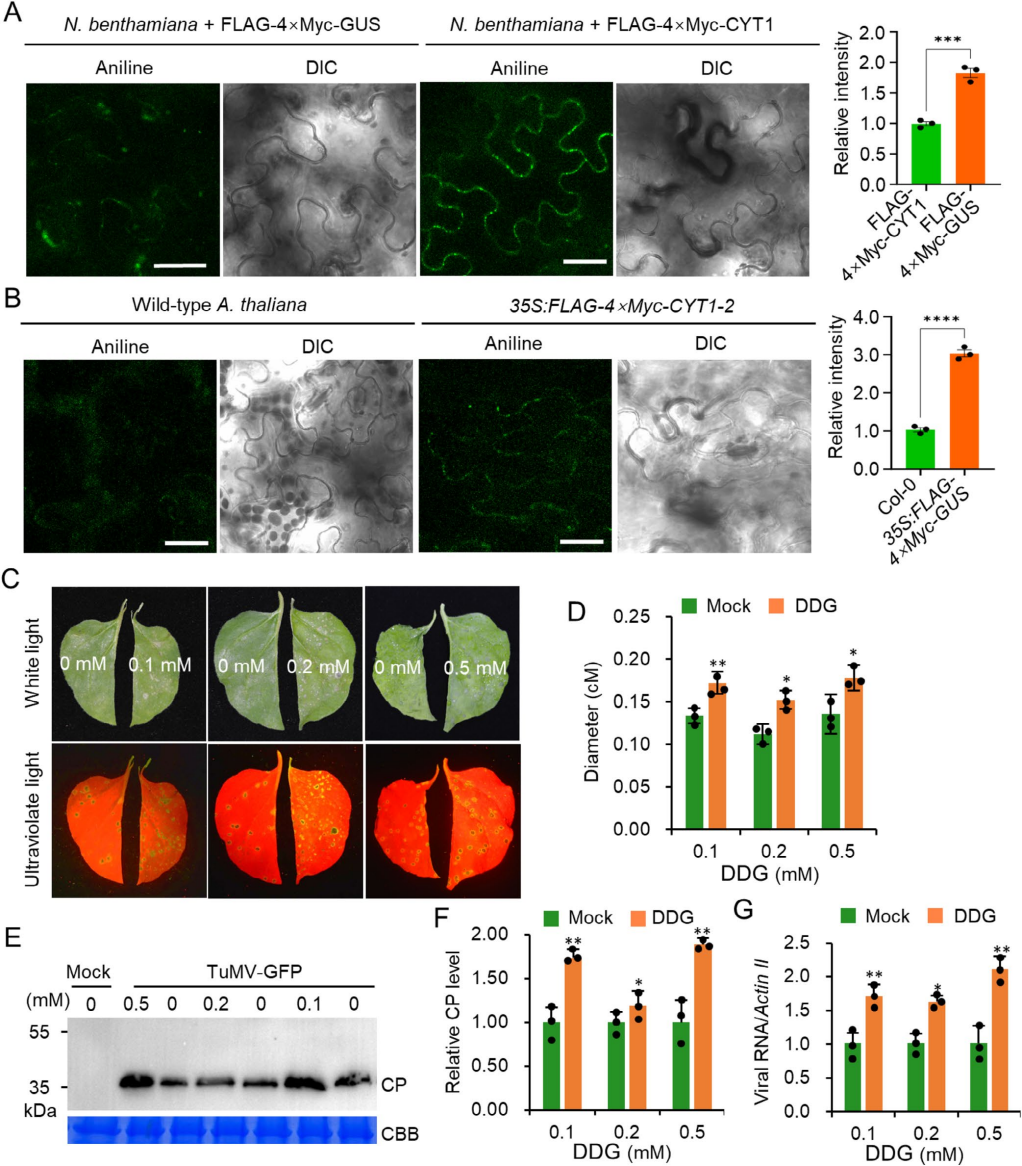
Overexpression of *CYT1* promotes callose deposition. (A) Confocal microscopy images of *Nicotiana benthamiana* epidermal cells expressing FLAG‐4 × Myc‐CYT1 or FLAG‐4 × Myc‐GUS (control) at 2 days post‐infiltration (dpi), stained with aniline blue. Scale bar: 50 μm. (B) Aniline blue‐stained callose in wild‐type (WT) and transgenic *Arabidopsis thaliana* leaf cells expressing FLAG‐4 × Myc‐CYT1. DIC (differential interference contrast) images show cell outlines. Scale bar: 50 μm. (C) Quantification of fluorescence intensity for (A) and (B). Mock treatment intensity was normalised to 1. (D) Phenotypes of *N. benthamiana* leaves treated with indicated 2‐deoxy‐d‐glucose (DDG) concentrations for 5 days post‐TuMV‐GFP inoculation under white and ultraviolet light. (E) Bar graph showing average TuMV lesion diameters in DDG‐treated *N. benthamiana* leaves at 5 dpi (*n* = 3 half‐leaves). (F) Immunoblot of TuMV coat protein (CP) accumulation. Each treatment includes five lesions. (G) Bar graph of relative CP levels normalised to mock treatment in the panel (F). (H) Reverse transcription‐quantitative PCR analysis of viral genome levels in mock‐ and DDG‐treated samples (*n* = 3), normalised to *Actin II*. In all panels, * and ** indicate *p* ≤ 0.05 and 0.01, respectively (Student's *t* test).

Plant viruses move between cells via plasmodesma (PD), and callose deposition has been shown to limit virus movement (Zavaliev et al. 2009; Li et al. 2012). Knockout of *CYT1* causes excessive callose deposition (Nickle and Meinke 1998). We hypothesised that CYT1 may regulate callose deposition. We transiently expressed FLAG‐4 × Myc‐CYT1 or FLAG‐4 × Myc‐GUS in *N. benthamiana* leaves under the CaMV 35S promoter via agroinfiltration and stained the agroinfiltrated leaf areas with aniline blue at 48 hpi. Confocal microscopy showed that transient expression of FLAG‐4 × Myc‐CYT1 in *N. benthamiana* leaves induced callose deposition at plasmodesma, as visualised by aniline blue staining (Figure 6A). Similarly, transgenic *Arabidopsis* overexpressing *CYT1* showed increased callose deposition compared to wild‐type plants (Figure 6B). To determine whether callose deposition limits TuMV infection, we treated TuMV‐infected *N. benthamiana* leaves with 2‐deoxy‐d‐glucose (DDG), an inhibitor of callose synthesis. We found that DDG treatment significantly increased the diameter of TuMV infection foci at 5 dpi (Figure 6C,D). Western blotting confirmed that DDG treatment significantly increased viral CP accumulation at 5 dpi (Figure 6E,F). RT‐qPCR showed that DDG treatment also significantly increased viral genome RNA accumulation (Figure 6G). Taken together, these results confirm that callose deposition plays a critical role in limiting TuMV infection.

## 3 Discussion

### 3.1 NIb Interacts With CYT1

Plant viruses have small genomes with extremely limited coding capacity and rely on multifunctional viral proteins that interact with host factors to facilitate infection and suppress host defences. Potyviral NIb has been shown to interact with many host proteins to promote viral infection and suppress host antiviral immunity (Martínez et al. 2023; Liu et al. 2023; Zhang et al. 2021; Li et al. 2018; Cheng et al. 2017). In this study, we identified 57 potential NIb‐interacting host factors, including CYT1, ARF2b, RUB1, and UEV1a. We confirmed the interactions between NIb and these factors using BiFC and Y2H assays. Thus, the potyviral NIb protein is an excellent example of a multifunctional viral protein.

Previously, we found that the salicylic acid (SA) plays an important role in inhibiting TuMV infection and that TuMV‐encoded NIb is able to target NPR1, the receptor of SA, to attenuate such antiviral immunity (Liu et al. 2023). Our findings suggest that NIb targets CYT1, a key component of AsA biosynthesis and *N*‐linked glycosylation, to suppress its antiviral function. NIb interacts with the C‐terminal LβH domain of CYT1, which may be involved in multimerisation and protein interaction, but not with the N‐terminal ROS catalytic domain. We found that NIb does not affect CYT1's subcellular localisation or stability reduces its cytosolic levels and AsA concentration. These results suggest that NIb may disrupt CYT1's interactions with other host factors, reducing its cytosolic availability and ASA synthesis for suppressing antiviral immunity.

The in vitro virus infection assay showed that tunicamycin treatment inhibits TuMV infection, suggesting that *N*‐linked glycosylation plays a role in TuMV infection. Although no potyviral protein has been reported to undergo *N*‐ or *O*‐linked glycosylation, the coat protein of plum pox virus (PPV) of the same genus as TuMV, can be *O*‐GlcN‐Acylated, another form of glycosylation that attaches single β‐*N*‐acetylglucosamine to serine (Ser) and/or threonine (Thr) side‐chains through an *O*‐β‐glycosidic bond (Martínez‐Turiño et al. 2018; Fernandez‐Fernandez et al. 2002; de Jesus Perez et al. 2006). Moreover, proteins of many animal and human viruses, for example, dengue, Zika, influenza, and human immunodeficiency viruses, as well as coronaviruses, can be glycosylated, and such glycosylation is necessary for viral infection (Feng et al. 2022). Therefore, it is also possible that NIb interacts with CYT1 to facilitate *N*‐linked glycosylation of itself or other viral proteins for robust infection.

### 3.2 AsA Inhibits TuMV Infection

CYT1 is involved in AsA biosynthesis and *N*‐linked glycosylation of proteins and cellulose, and its antiviral function may be mediated by multiple mechanisms, including interference with viral replication and regulation of host immune responses. AsA, as an antioxidant, plays a crucial role in reducing oxidative damage during immune priming. Our virus infection assays showed that AsA treatment significantly reduced TuMV lesion size and viral CP and genomic RNA accumulation, suggesting that AsA inhibits TuMV infection through direct or indirect mechanisms. AsA may enhance the plant's antioxidant defence system, or it may regulate the immune response to increase resistance to viruses. Inhibition of *N*‐linked glycosylation also reduced TuMV infection, suggesting that *N*‐linked glycosylation may either directly facilitate TuMV infection or indirectly inhibit virus infection by promoting AsA biosynthesis. Therefore, it is possible that CYT1 may play dual roles during TuMV infection: *N*‐linked glycosylation may transiently support virus replication; the net effect of *CYT1* overexpression is antiviral due to enhanced AsA and callose deposition. Further studies are needed to elucidate the precise mechanisms of *N*‐linked glycosylation in virus infection and plant antiviral immunity and how CYT1 balances these two functions during virus infection.

### 3.3 CYT1‐Induced Callose Deposition Limits TuMV Infection

Our data showed that transient expression of *CYT1* in *N. benthamiana* or transgenic overexpression of *CYT1* induced callose deposition at PD, although the effect of *CYT1* knockdown or knockout on TuMV infection is still lacking. Moreover, our results showed that inhibition of callose synthesis enhanced TuMV infection. Callose is an important component of the plant cell wall, and its deposition at PD can decrease its cavity size, which prevents virus spread (Tilsner et al. 2014). CYT1‐induced callose deposition may be a key mechanism of antiviral immunity in plants (Ge et al. 2024; Huang et al. 2023). Interestingly, knockout of *CYT1* also causes excessive callose deposition (Nickle and Meinke 1998). Knockout of *CYT1* causes cellulose deficiency, which may trigger compensatory callose deposition as a stress response (Lukowitz et al. 2001). Knockout of *CYT1* can lead to excessive mannose accumulation and its potential diversion to mannitol. Both mannose and mannitol act as cellular stressors, which may induce callose synthesis via stress‐activated signalling pathways (Zhifang and Loescher 2003; Zhao et al. 2020; Sangi et al. 2023). Alternatively, CYT1‐mediated *N*‐linked glycosylation modulates cellulose/callose balance, influencing plasmodesmatal trafficking. The exact molecular mechanisms by which CYT1 promotes callose deposition and AsA accumulation require further investigation.

## 4 Experimental Procedures

### 4.1 Plant Materials and Growth Conditions

Wild‐type *A. thaliana* ecotype Columbia‐0 (Col‐0) and *N. benthamiana* seedlings were grown in pots in a plant growth chamber at 23°C under a 16 h:8 h (light:dark) photoperiod. Transgenic *35S:FLAG‐4 × Myc‐CYT1* plants were generated using the floral dip method in a wild‐type Col‐0 genetic background (Liu et al. 2023).

### 4.2 Plasmid Construction

The TuMV infectious clone expressing free GFP (TuMV‐GFP) and an additional mCherry‐tagged 6K2 (TuMV‐6K2mCherry) between P1 and HcPro cistrons was previously described (Lellis et al. 2002; Cotton et al. 2009) as was the TuMV movement‐defective mutant TuMV‐GFP/ΔCP (Dai et al. 2020). The full‐length coding sequences of *CYT1* (AT2G39770), *RUB1* (AT1G31340), *UEV1a* (AT1G23260), *EML1* (AT3G12140), and *CCA1* (AT2G46830) were amplified from wild‐type *A. thaliana* Col‐0 cDNA using Phanta Flash Master Mix (Vazyme) with the primers listed in Table S3. Amplified DNA fragments were cloned into a modified pDONR207 vector and verified by Sanger sequencing. Entry plasmids were recombined into destination vectors using the Gateway LR Clonase II Enzyme Mix (ThermoFisher Scientific). The vectors pEarleyGate‐101 and pBA‐FLAG‐4 × Myc‐DC (Earley et al. 2006; Zhu et al. 2011) were used to construct C‐terminal YFP‐ and N‐terminal FLAG‐4 × Myc‐tagged recombinant proteins, respectively. The pGBKT7‐DEST and pGADT7‐DEST were used for the Y2H assays (Lu et al. 2010), while pEarleyGate201‐YN and pEarleyGate202‐YC were used for the BiFC assays (Lu et al. 2010).

### 4.3 Mechanical Inoculation

Mechanical inoculation was performed as described previously (Liu et al. 2023). In brief, TuMV‐infected *N. benthamiana* leaf tissues were homogenised in 10 mM phosphate buffer (pH 7.5) and used to inoculate leaves of *A. thaliana* seedlings or detached *N. benthamiana* leaves predusted with 600‐mesh carborundum. After 2–3 min, leaves were rinsed with distilled water and covered with moistened paper towels. *A. thaliana* plants were returned to the growth chamber for symptom development, while detached *N. benthamiana* leaves were placed in Petri dishes on moistened paper, sealed with Parafilm, and incubated under light for lesion development.

### 4.4 Agroinfiltration

*Agrobacterum tumefaciens* GV3101 carrying the appropriate plasmids was grown overnight in liquid Luria Bertani medium supplemented with antibiotics at 28°C. Bacterial cells were pelleted by centrifugation at 6000 *g* , washed once with the infiltration buffer (10 mM MES, pH 5.6; 10 mM MgCl_2_; 100 μM acetosyringone), and resuspended in the same buffer. After incubation at room temperature for 2 h, the bacteria were infiltrated into *N. benthamiana* leaves using a needleless syringe at an optical density at 600 nm (OD_600_) of 0.4 for transient expression or OD_600_ of 0.2 for virus inoculation.

### 4.5 Immunoprecipitation and Mass Spectrometry

Total protein was extracted from 1 g of TuMV‐GFP‐infected or healthy *A. thaliana* Col‐0 leaf tissue by grinding in liquid nitrogen and homogenising in 10 mL immunoprecipitation (IP) buffer (20 mM Tris‐HCl, pH 7.5; 150 mM NaCl; 1% NP‐40; 1/2 tablet of Complete Protease Inhibitor [Roche]). After incubating on ice for 20 min, the lysate was filtered through two layers of Miracloth (Merck Millipore), sonicated on ice (10% power, 10 s pulses, 1 min intervals for 20 min), and centrifuged at 6000 *g* for 20 min at 4°C. Supernatants were incubated with 30 μL of Chromotek GFP‐Trap agarose resin (Proteintech) for 2 h on ice. Beads were washed five times with IP buffer and either used for western blotting or sent to Bioprofile technology (Shanghai) for MS/MS analysis. Proteins were digested with trypsin (Promega) and analysed using an Easy nLC 1200 mass spectrometer (ThermoFisher Scientific). Peptides were identified against the Arabidopsis UniProt database (accessed on Aug 10, 2022) and TuMV protein sequences using Mascot 2.5 (Matrix Science) with fixed modifications: carbamidomethyl, variable modification: Gln‐pyro‐Glu, oxidation; peptide mass tolerance: ±15 ppm; fragment mass tolerance: ±20 mmu; max missed cleavages: 2). The protein score is −10log_10_(*P*), where *P* is the absolute probability (individual scores > 46 indicate identity or extreme homology (*p* < 0.05), while emPAI is quantitation for the proteins in a mixture based on protein coverage by the peptide matches in a database search result.

### 4.6 Confocal Microscopy

Confocal imaging was performed using a Leica TCS SP8 laser microscope as previously described (Liu et al. 2023). YFP was excited at 488 nm (7% energy), and emission was collected at 510–560 nm. Aniline blue was excited at 405 nm (15% energy), with emission detected at 420–500 nm.

### 4.7 Y2H Assay

Y2HGold yeast cells were transformed using a yeast transformation kit (Coolaber). Transformants were plated on synthetic defined (SD) medium lacking Leu and Trp (−LW) and incubated at 30°C until colonies formed. Colonies were serially diluted in distilled water, plated on SD medium lacking Leu, Trp, His, and Ade (−LWHA), and incubated at 30°C for 3–4 days before imaging.

### 4.8 Chemical Treatment and Callose Staining

AsA, tunicamycin, and DDG were dissolved in distilled water and sprayed onto *N. benthamiana* leaves. For callose staining, aniline blue (0.1% in deionised water, mixed 2:3 with glycerol) was infiltrated into leaves using a needleless syringe. Stained tissues were imaged under a Leica TCS SP8 microscope 30 min post‐infiltration.

### 4.9 Immunoblotting

Western blotting was performed as previously described (Liu et al. 2023). Primary antibodies included rabbit anti‐GFP N‐terminal domain (1:5000; Sigma‐Aldrich, G1544), rabbit anti‐Myc tag (1:2000; Abcam, ab32072), and polyclonal antibodies against TuMV CP and NIb that were produced by ABclonal Biotechnology Co. Ltd. and were described previously and were used at 1:1000 dilution (Liu et al. 2023). Goat anti‐rabbit IgG horseradish peroxidase conjugate antibodies (catalogue no. DC03L; Sigma‐Aldrich) were used at 1:5000 dilution.

### 4.10 Measure of AsA Concentration

The concentration of AsA was measured using the ascorbic acid (AsA) content detection kit (Solarbio, bc1230) according to the instructions of the manufacturer.

### 4.11 Quantitative Analysis

Quantitative analyses of *Arabidopsis* plants and *N. benthamiana* leaves were performed as previously described (Liu et al. 2023). Briefly, UV images were split into red, green, and blue channels using the Image‐Adjust‐Threshold function in Fiji (Schindelin et al. 2012). Total and virus‐infected leaf area were quantified using the Analyse Particles function (size: 100‐infinity; circularity: 0.0–1.0). Pseudocolour images were generated with the ROI manager. For confocal images, aniline blue fluorescence (≥ 5 pixels; circularity 0.2–1.0) was quantified using particle analysis. Western blot bands were analysed with GelAnalyzer v. 19.1 (http://www.gelanalyzer.com/).

### 4.12 Statistical Analyses

Data are presented as mean ± SD. Statistical significance was determined by two‐tailed Student's *t* test using Microsoft Excel.

## Author Contributions

**Xue Jiang:** investigation (equal), visualisation, writing – original draftpreparation. **Yuting Wang:** investigation (equal), formal analysis, visualisation. **Yu Zhao:** investigation (equal), visualisation, writing – original draftpreparation (equal). **Xayvangye Korxeelor:** validation, data curation. **Wenqian Fan:** data curation, validation. **Xinyue Fan:** datacuration, validation. **Yong Li:** software, resources. **Xiaoxia Wu:** resources, validation. **Xueping Zhou:** resources. **Fangfang Li:** methodology. **Xiaoyun Wu:** resources, writing – review and editing. **Weiqin Ji:** conceptualization (supporting), project administration (supporting), writing – review and editing. **Xiaofei Cheng:** conceptualization, datacuration, funding acquisition, project administration, supervision, writing – review and editing.

## Conflicts of Interest

The authors declare no conflicts of interest.

## Supporting information


**Figure S1.** Bar graph of relative viral genome levels in *Nicotiana benthamiana* leaves infected by TuMV‐GFP/ΔCP and treated with 10 or 50 mM ascorbic acid (AsA) at 5 days post‐infiltration. Reverse transcription–quantitative PCR data were normalised to *Actin II*. * and ** indicate *p* ≤ 0.05 and 0.01, respectively (Student’s *t* test).

**Figure S2.** Western blotting for the expression of CYT1‐YFP, FLAG‐4 × Myc‐NIb, and FLAG‐4 × Myc‐GUS.

**Figure S3.** (A) Immunoblot of CYT1‐YFP and YFP‐CYT1 accumulation in the presence of FLAG‐4 × Myc‐NIb or FLAG‐4 × Myc‐GUS (control). (B) Confocal microscopy images of *Nicotiana benthamiana* epidermal cells coexpressing NIb‐RFP and CYT1‐YFP or YFP (control) at 2 days post‐infiltration. Scale bar: 50 μm.

**Figure S4.** Bar graph of relative ascorbic acid (AsA) levels in *Nicotiana benthamiana* leaves infected by TuMV‐GFP at 0, 3, 6, and 12 days post‐inoculation.

**Table S1.** TuMV peptides identified by tandem mass spectrometry.

**Table S2.** NIb‐interacting candidates identified by tandem mass spectrometry.

**Table S3.** Primers used in this study.

## Data Availability

The data that support the finding of this study are available in the Supporting Information of this article.
