# Correction to ‘Cytokinesis‐Defective 1 (CYT1) Positively Regulates Plant Antiviral Immunity by Promoting Callose Deposition and Ascorbic Acid Biosynthesis’

**DOI:** 10.1111/mpp.70156

**Published:** 2025-09-15

Jiang X, Y Wang, Y Zhao, X Korxeelor, W Fan, X Fan, Y Li, X Wu, X Zhou, F Li, X Wu, W Ji, X Cheng. 2025. “Cytokinesis‐Defective 1 (CYT1) Positively Regulates Plant Antiviral Immunity by Promoting Callose Deposition and Ascorbic Acid Biosynthesis.” Molecular Plant Pathology 26: e70126.

In the figure legend of Figure 6, panel C actually refers to the bar graph of panels A and B (not labelled in Figure 6), which cause the subsequent panels C–G to become D–H in the figure legends.

Here is the correct figure legend for Figure 6:

Overexpression of *CYT1* promotes callose deposition. (A) Confocal microscopy images of *Nicotiana benthamiana* epidermal cells expressing FLAG‐4 × Myc‐CYT1 or FLAG‐4 × Myc‐GUS (control) at 2 days post‐infiltration (dpi), stained with aniline blue. Scale bar: 50 μm. The right panel shows the quantification of fluorescence intensity. Mock treatment intensity was normalised to 1. (B) Aniline blue‐stained callose in wild‐type (WT) and transgenic *Arabidopsis thaliana* leaf cells expressing FLAG‐4 × Myc‐CYT1. DIC (differential interference contrast) images show cell outlines. Scale bar: 50 μm. The right panel shows the quantification of fluorescence intensity. Mock treatment intensity was normalised to 1. (C) Phenotypes of *N. benthamiana* leaves treated with indicated 2‐deoxy‐d‐glucose (DDG) concentrations for 5 days post‐TuMV‐GFP inoculation under white and ultraviolet light. (D) Bar graph showing average TuMV lesion diameters in DDG‐treated *N. benthamiana* leaves at 5 dpi (*n* = 3 half‐leaves). (E) Immunoblot of TuMV coat protein (CP) accumulation. Each treatment includes five lesions. (F) Bar graph of relative CP levels normalised to mock treatment in the panel (E). (G) Reverse transcription‐quantitative PCR analysis of viral genome levels in mock‐ and DDG‐treated samples (*n* = 3), normalised to *Actin II*. In all panels, * and ** indicate *p* ≤ 0.05 and 0.01, respectively (Student's *t* test).

We apologise for this error.

