# Supplementary figures and images for: Cytokinesis‐Defective 1 (CYT1) Positively Regulates Plant Antiviral Immunity by Promoting Callose Deposition and Ascorbic Acid Biosynthesis
Source: Mol Plant Pathol. 2025 Jul 9;26(7):e70126. doi: 10.1111/mpp.70126 (PMC12241708; doi:10.1111/mpp.70126)

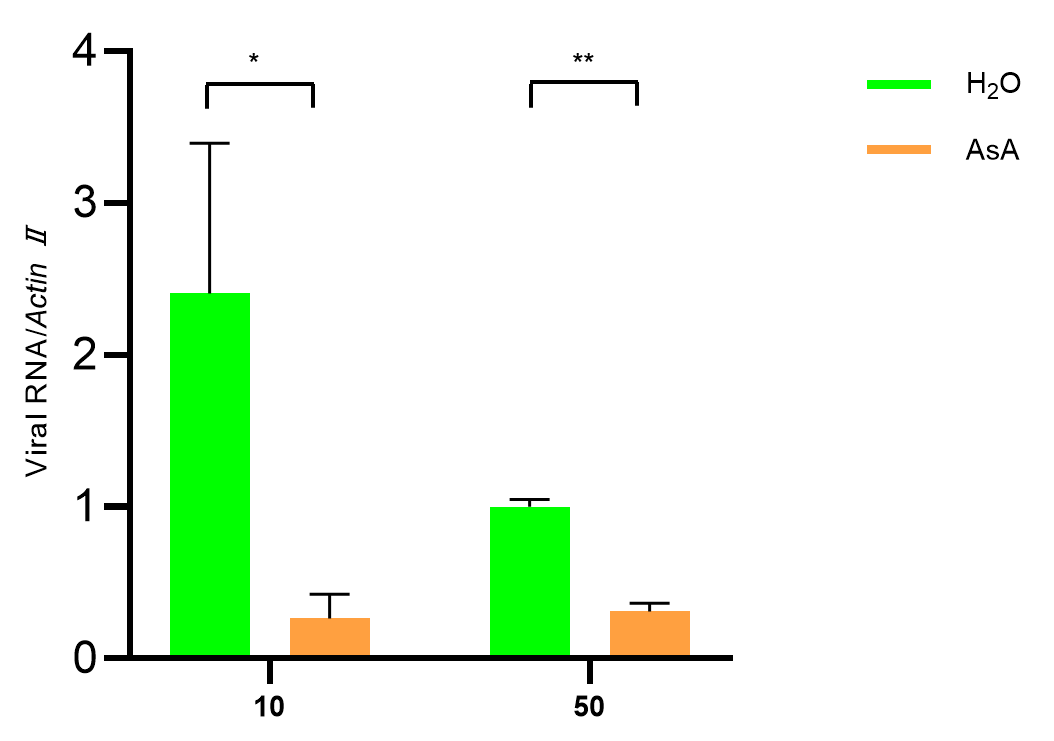

Supplement: Supplementary file 1 — Figure S1. Bar graph of relative viral genome levels in Nicotiana benthamiana leaves infected by TuMV‐GFP/ΔCP and treated with 10 or 50 mM ascorbic acid (AsA) at 5 days post‐infiltration. Reverse transcription–quantitative PCR data were normalised to Actin II. * and ** indicate p ≤ 0.05 and 0.01, respectively (Student’s t test). [file MPP-26-e70126-s006.tif]

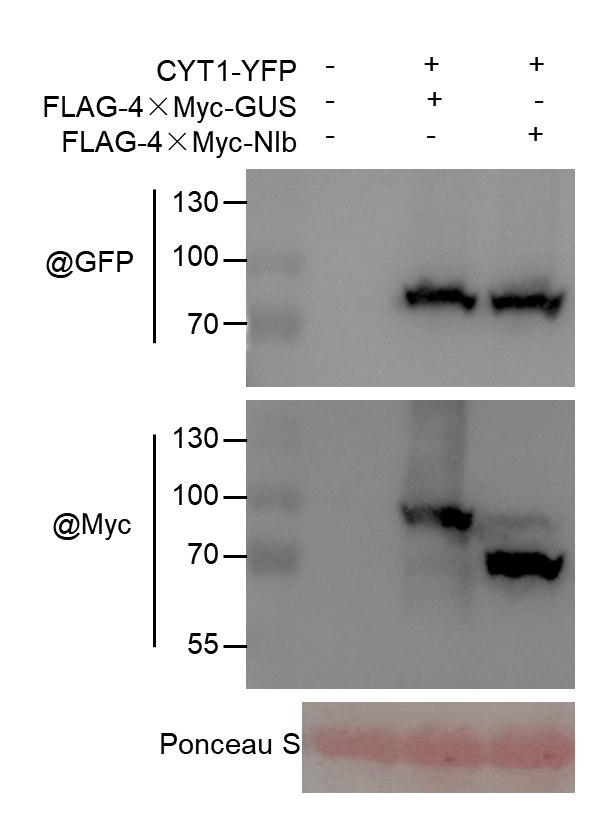

Supplement: Supplementary file 2 — Figure S2. Western blotting for the expression of CYT1‐YFP, FLAG‐4 × Myc‐NIb, and FLAG‐4 × Myc‐GUS. [file MPP-26-e70126-s003.tif]

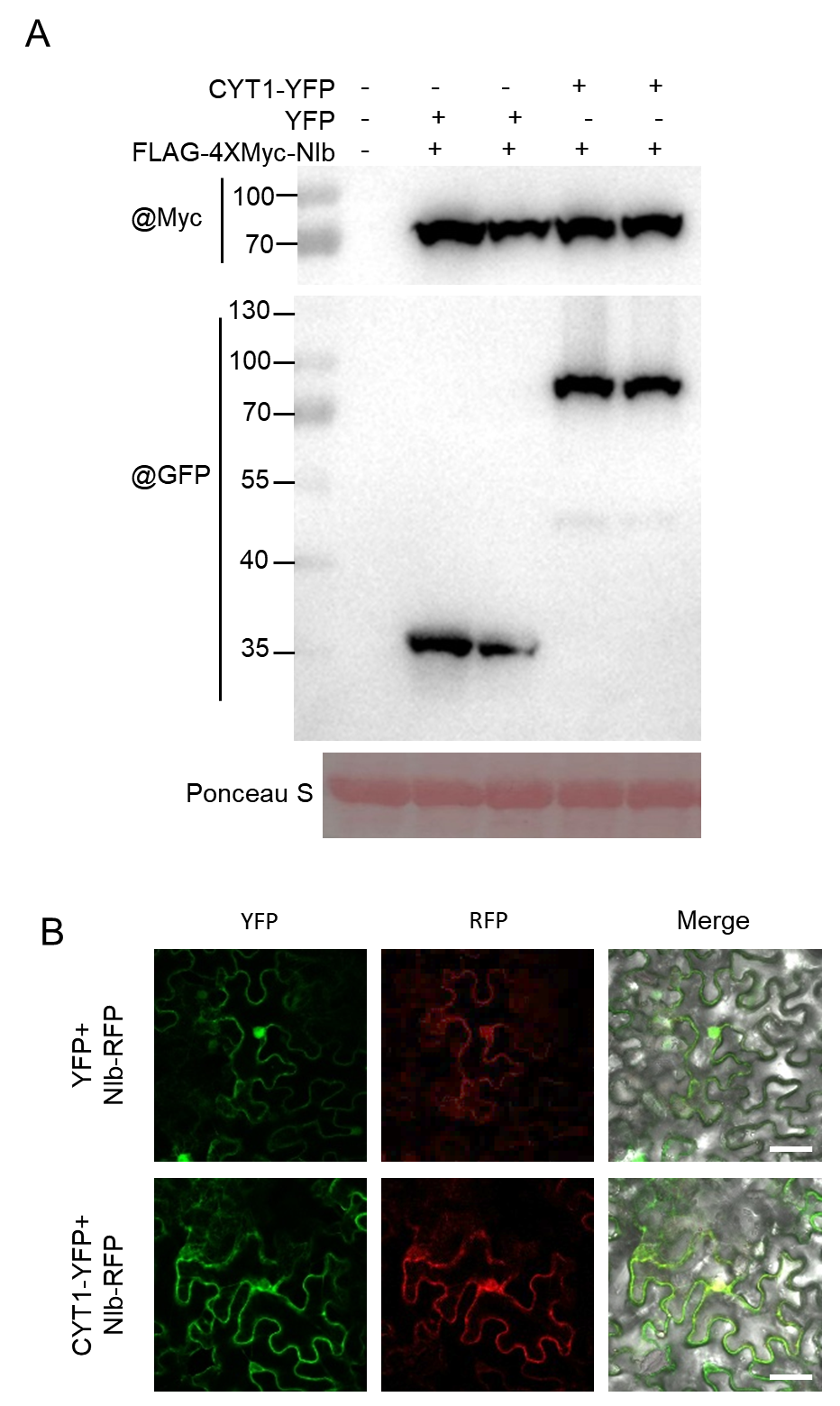

Supplement: Supplementary file 3 — Figure S3. (A) Immunoblot of CYT1‐YFP and YFP‐CYT1 accumulation in the presence of FLAG‐4 × Myc‐NIb or FLAG‐4 × Myc‐GUS (control). (B) Confocal microscopy images of Nicotiana benthamiana epidermal cells coexpressing NIb‐RFP and CYT1‐YFP or YFP (control) at 2 days post‐infiltration. Scale bar: 50 μm. [file MPP-26-e70126-s001.tif]

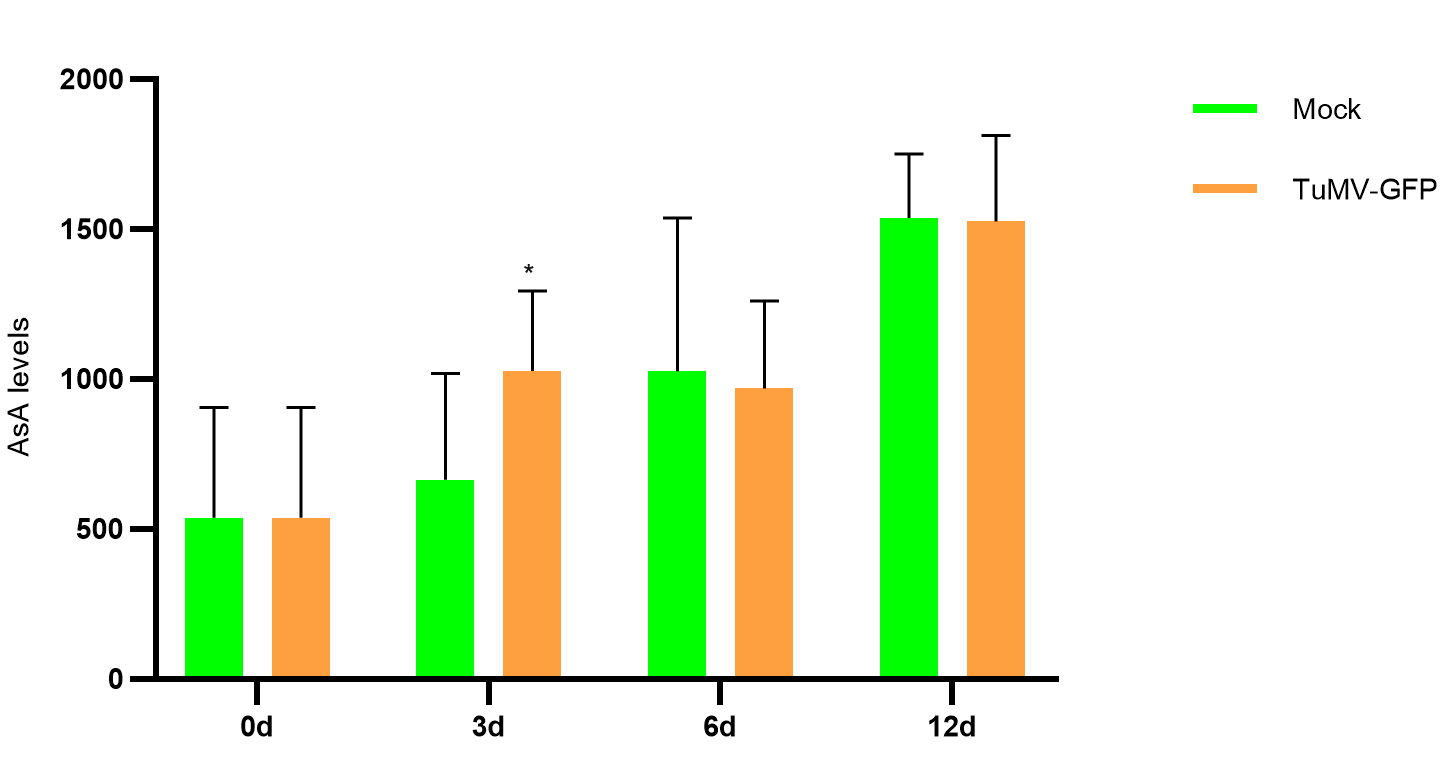

Supplement: Supplementary file 4 — Figure S4. Bar graph of relative ascorbic acid (AsA) levels in Nicotiana benthamiana leaves infected by TuMV‐GFP at 0, 3, 6, and 12 days post‐inoculation. [file MPP-26-e70126-s007.tif]
