# Supplementary material for: Cytokinesis‐Defective 1 (CYT1) Positively Regulates Plant Antiviral Immunity by Promoting Callose Deposition and Ascorbic Acid Biosynthesis
Source: Mol Plant Pathol. 2025 Jul 9;26(7):e70126. doi: 10.1111/mpp.70126 (PMC12241708; doi:10.1111/mpp.70126)
Supplement: Supplementary file 7 — Table S3. Primers used in this study. [file MPP-26-e70126-s004.docx]

**Supplementary Table 3 Primers used in this study.**

| Primer Name | Sequence (5ʹ-3ʹ) |
| --- | --- |
| pDonR207-CYT1-F | GTACAAAAAAGCAGGCTTCATGAAGGCACTCATTCTTGTTGGAG |
| pDonR207-CYT1-R | CAAGAAAGCTGGGTCCCGACGCATCACTATCTCTGGCTTCAAGATGTTT |
| pDonR207-CYT1-C1-F | GTACAAAAAAGCAGGCTTCATGAAGGCACTCATTCTTGTTGGAGG |
| pDonR207-CYT1-C1-R | CAAGAAAGCTGGGTCCCGACGACTGGTTAATTTGGCAGGAGATTTCTTCC |
| pDonR207-CYT1-C2-F | GTACAAAAAAGCAGGCTTCGGGCCACACATAGTTGGGAATGTTC |
| pDonR207-CYT1-C2-R | CAAGAAAGCTGGGTCCCGACGCATCACTATCTCTGGCTTCAAGATGTTTG |
| ACTIN2-RTF | CCGGTATTGTGCTGGATTCT |
| ACTIN2-RTR | TTCTCGATGGAAGAGCTGGT |
| TuMV CP-RTF | CAGGTTTGACAGACGAGCAA |
| TuMV CP-RTR | CCAGAGGTTCCAGCGTTTAC |
| PGAD-R | AGATGGTGCACGATGCACAG |
| PGBK-R | TAAGAGTCACTTTAAAATTTGTAT |
| 4Myc-F | gctcatttctgaagaggact |
| pDonR207-RUB1-dorF | GTACAAAAAAGCAGGCTTCATGCAGATCTTCGTCAAAACCCTC |
| pDonR207-RUB1-dorR | CAAGAAAGCTGGGTCCCGACGGAGAAGACCAAAACCACCCCTAAG |
| pDonR207-ACC1-dorF | GTACAAAAAAGCAGGCTTCATGGTTGATCAAGTTCAGCACCC |
| pDonR207-ACC1-dorR | CAAGAAAGCTGGGTCCCGACGGGCACCTCCTGATCCGTACTTC |
| pDonR207-EML1-dorF | GTACAAAAAAGCAGGCTTCATGGAGACACAAATTCATCAACTTGAGC |
| pDonR207-EML1-dorR | CAAGAAAGCTGGGTCCCGACGTCCCTGTGGCATTGGATGATCATG |
| pDonR207-UEV1A-dorF | GTACAAAAAAGCAGGCTTCATGGATGATGCTGATGATATTTATATGC |
| pDonR207-UEV1A-dorR | CAAGAAAGCTGGGTCCCGACGCATCACACAACATTTAGCTGGTCC |
